# SEnSCA: Identifying possible ligand‐receptor interactions and its application in cell–cell communication inference

**DOI:** 10.1111/jcmm.18372

**Published:** 2024-05-15

**Authors:** Liqian Zhou, Xiwen Wang, Lihong Peng, Min Chen, Hong Wen

**Affiliations:** ^1^ School of Life Sciences and Chemistry Hunan University of Technology Hunan China; ^2^ School of Computer Science Hunan Institute of Technology Hengyang China; ^3^ School of Computer Science Hunan University of Technology Hunan China

**Keywords:** convolutional neural network, intercellular communication, ligand‐receptor interaction, multi‐head attention mechanism, stacking ensemble

## Abstract

Multicellular organisms have dense affinity with the coordination of cellular activities, which severely depend on communication across diverse cell types. Cell–cell communication (CCC) is often mediated via ligand‐receptor interactions (LRIs). Existing CCC inference methods are limited to known LRIs. To address this problem, we developed a comprehensive CCC analysis tool SEnSCA by integrating single cell RNA sequencing and proteome data. SEnSCA mainly contains potential LRI acquisition and CCC strength evaluation. For acquiring potential LRIs, it first extracts LRI features and reduces the feature dimension, subsequently constructs negative LRI samples through K‐means clustering, finally acquires potential LRIs based on Stacking ensemble comprising support vector machine, 1D‐convolutional neural networks and multi‐head attention mechanism. During CCC strength evaluation, SEnSCA conducts LRI filtering and then infers CCC by combining the three‐point estimation approach and single cell RNA sequencing data. SEnSCA computed better precision, recall, accuracy, F1 score, AUC and AUPR under most of conditions when predicting possible LRIs. To better illustrate the inferred CCC network, SEnSCA provided three visualization options: heatmap, bubble diagram and network diagram. Its application on human melanoma tissue demonstrated its reliability in CCC detection. In summary, SEnSCA offers a useful CCC inference tool and is freely available at https://github.com/plhhnu/SEnSCA.

## 1 INTRODUCTION

Multicellular organisms have close linkages with the coordination of cellular activities. These activities severely depend on cell–cell communication (CCC) across diverse cell types.^1^, ^2^, ^3^, ^4^ Cells accomplish their functions by communicating with other cells. Consequently, CCC is fundamental to numerous biological processes including cellular proliferation, migration, homeostasis and fate decisions. It plays key roles in accomplishing cellular functions and maintaining tissue homeostasis.

In particular, cancers are complex heterocellular systems composed of epithelial cancer cells, stromal fibroblasts and immune cells. Within tumour microenvironment, CCC significantly changes due to the difference of the extracellular matrix, and further cause cancer progression and influences the responses to therapies. Elucidating CCC helps enrich our understanding about the mechanisms of cancer development and metastasis and provides insight into new therapeutic options.^5^

CCC occurs when the sender cell transmits signals to the receiver cell via signalling molecules. These signalling molecules include ligands, receptors, structural proteins, junction proteins, ions, metabolites and so on. A typical signalling event begins with interactions between diverse proteins, such as ligand‐receptor interactions (LRIs), where the receiver cell activates downstream signalling via interaction with homologous receptors.^3^, ^6^ Consequently, CCC can be regarded as a one‐to‐one interaction between transmitting and receiving proteins. That is, complex CCC begins with LRIs, which trigger specific cellular signalling pathways. Thus, LRI analysis is the basis for dissecting cellular behaviour and activation corresponding to neighbouring cells.^7^

Recently, LRI‐mediated CCC identification has been the most frequent scenario for computational CCC analysis.^8^ The knowledge about LRIs is usually obtained from numerous protein–protein interaction (PPI) data sources.^9^ Thus, many computational methods have been used to find potential PPIs. However, compared to PPI networks, an overall human cell‐surface interactome still remains lacking. Several recent researches have been devoted to screening LRIs via high‐throughput experiments, providing valuable resources for intercellular communication inference.^10^ However, high‐throughput experimental techniques have limitations due to high time consuming and cost. Thus, several computational methods have been exploited to predict LRIs. For example, NicheNet^11^ pinpointed ligand‐target gene linkages between communicating cells by integrating their gene expression with prior information based on the Personalized PageRank algorithm. CellPhoneDB^12^ is a novel repository including ligands, receptors and LRIs. scTenifoldXct^13^ embedded ligand and receptor expression in communicating cells into a unified latent space by minimizing their distance based on neural network. CellEnBoost^14^ and CellComNet^6^ are two ensemble deep learning algorithms by combining LRI feature extraction, feature selection and classification. CellDialog^4^ capitalized on a KTBoost‐based LRI identification framework. GCNG found novel extracellular interacting gene pairs based on graph convolutional neural networks (CNNs).^15^ Giotto^16^ exploited a comprehensive, open‐source, flexible and robust framework for analysing LRIs.

The above methods effectively found a few LRIs, but they were primarily contingent upon the specific research objectives and the available data types. With the development of sequencing technologies, single cell RNA sequencing (scRNA‐seq) provides numerous gene expression information for single cells.^17^ Here, we developed an LRI prediction method named SEnSCA for intercellular communication analysis by combining scRNA‐seq data.

## 2 MATERIALS AND METHODS

### 2.1 The SEnSCA pipeline

**FIGURE 1:**
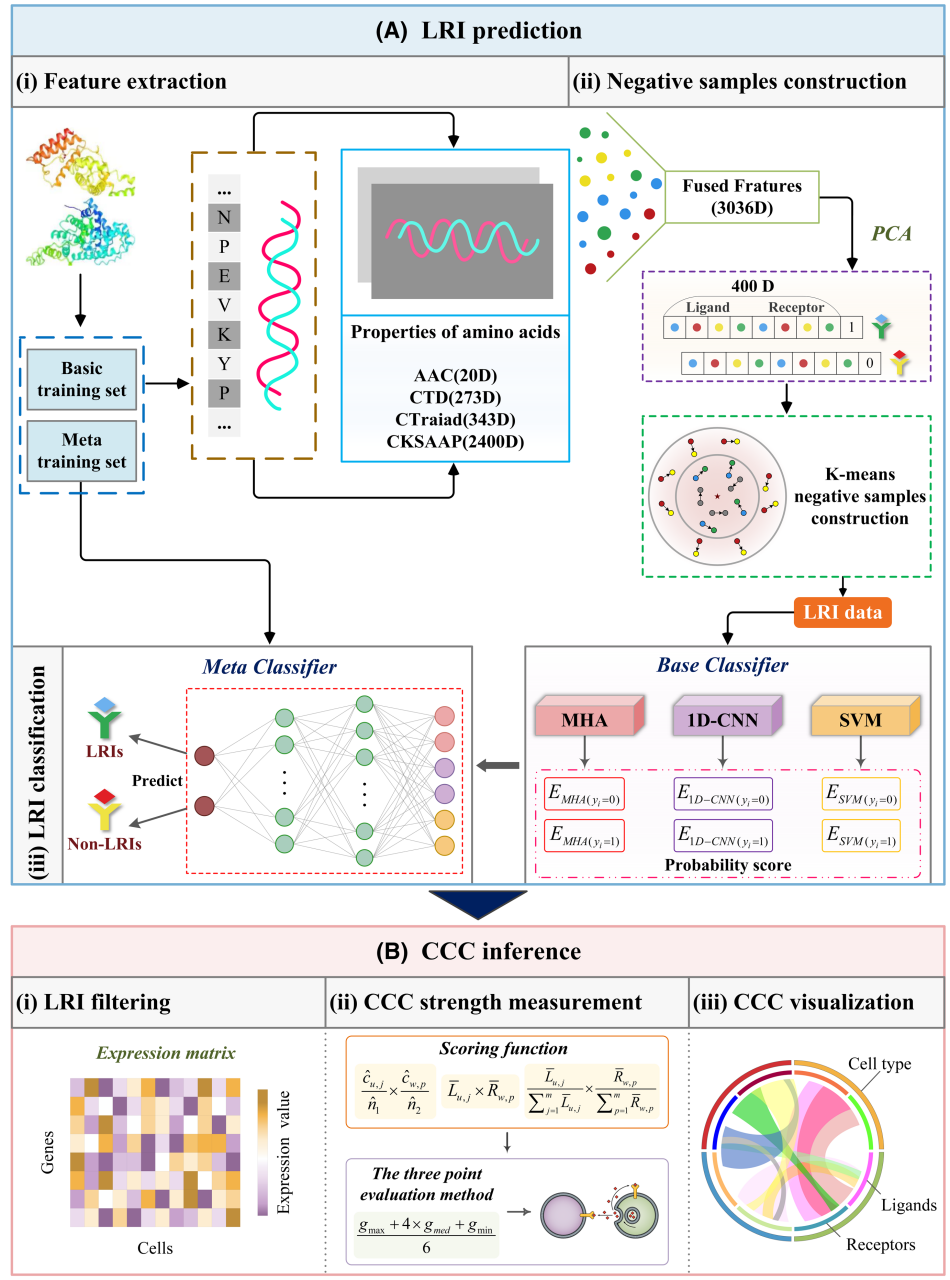
The pipeline for CCC prediction framework SEnSCA. (A) LRI prediction. LRIs are predicted through the following steps: (i) LRI feature extraction using iFeature; (ii) Construction of negative LRI samples through K‐means clustering; and (iii) LRI classification using a Stacking ensemble consisting of support vector machine, 1D‐convolutional neural networks and multi‐head attention mechanism. (B) CCC inference. CCC is inferred by incorporating the following processes: (i) LRI filtering based on interaction probability threshold and scRNA‐seq data; (ii) CCC strength measurement based on the three‐point evaluation approach; and (iii) CCC visualization through heatmap and network views.

We proposed a novel competitive model named SEnSCA for inferring CCC by integrating single cell transcriptomics and proteome data. The framework of SEnSCA is illustrated in Figure 1. The detailed procedures are as follows: LRI prediction. We predict LRIs by feature extraction, dimensionality reduction, negative sample construction and LRI classification. CCC inference. First, we conduct filtering for known and predicted LRIs based on a given threshold and scRNA‐seq data. Second, we use cell expression, expression product and specific expression to calculate the LRI‐mediated CCC score between two cell types. Third, the three‐point evaluation approach is used to calculate the final CCC score by integrating the results from the above three approaches. Finally, we visualize the results through heatmap, bubble diagram and network diagram.

### 2.2 Data preparation

We employed four distinct LRI datasets provided by Refs. [6, 14] to assess the SEnSCA LRI prediction ability. Dataset 1 and Dataset 2 were from a comprehensive LRI database CellTalkDB^18^ and encompass 3398 human LRIs between 812 ligands and 780 receptors and 2033 mouse LRIs between 650 ligands and 588 receptors after preprocessing. Dataset 3 was from Ref. [19] and includes 2009 mouse LRIs between 574 ligands and 559 receptors orthologous to human ones from Ref. [20] after preprocessing. Dataset 4, which was extracted from iRefIndex, Pathway Commons and BioGRID by Ref. [21] comprises 6638 LRIs between 1129 ligands and 1335 receptors after preprocessing.

### 2.3 Feature extraction and dimensionality reduction

To meticulously delineate each LR pair, we extract the sequence information of ligands and receptors from the UniProt database^22^ and use iFeature (iFeature Web Server (monash.edu)) for LRI feature extraction. These features encompass 20 amino acid composition, 2400 composition of k‐spaced amino acid pairs, 273 composition, transition and distribution, and 343 conjoint triad. Finally, each ligand or receptor is depicted as a 3036‐dimensional vector.

Since high‐dimensional data can aggravate the burden of model training and demand more storage space, we employ principal component analysis for dimension reduction. Consequently, each ligand or receptor is delineated as a d‐dimensional vector and each LR pair is represented as a 2d‐dimensional vector after concatenation operation.

For an LRI dataset $A=\left\{X,Y\right\}$ with n ligand‐receptor (LR) pairs, let $x_{i}$ and $y_{i}$ represent the i‐th LR pair with 2d‐dimensional features (i.e. the i‐th training sample) and its corresponding label, $y_{i}=1$ if the pair is interacting, and 0 otherwise. We aim to classify LR pairs without known labels.

### 2.4 Negative sample construction

There are a limited number of positive LRIs, a large amount of unlabeled LR pairs and there are not negative LRIs on four LRI datasets. However, the quality and quantity of negative samples directly impact the performance of the classification model. Randomly select negative sample from unlabeled LR pairs can potentially result in misclassification in LRI prediction. To establish a more accurate classification model, we exploit a K‐means clustering‐based negative LRI selection approach.

Given an LRI dataset with z positive samples, let the set of positive LRIs be denoted as $B\in {\mathbb{R}}^{z\times 2d}$, and the i‐th positive sample is represented as $\left[b_{i}^{1},b_{i}^{2},\ldots ,b_{i}^{2d}\right]$. First, we take the existing positive LRIs as one class and compute its centroid $\left[a^{1},a^{2},\ldots ,a^{2d}\right]$. Next, as shown in Equation (1), we calculate the Euclidean distance Dis between each positive LRI sample and this centroid and find the smallest distance minDis and largest distance maxDis between all positive LRIs and the centroid. Subsequently, as depicted in Equations ((2), (3), (4)), we compute the difference disLen between minDis and maxDis and then construct a range with the lower bound minRange and the upper bound maxRange according to a predefined threshold pre.

(1) $$\mathrm{Dis}=\sqrt{{\left(b_{i}^{1}-a^{1}\right)}^{2}+{\left(b_{i}^{2}-a^{2}\right)}^{2}+\cdots +{\left(b_{i}^{2d}-a^{2d}\right)}^{2}}$$

(2) $$\begin{array}{c} \text{disLen}=\text{maxDis}-\text{minDis} \end{array}$$

(3) $$\begin{array}{c} \text{minRange}=\text{minDis}+\left(1-\mathrm{pre}\right)\times 0.5\times \text{disLen} \end{array}$$

(4) $$\begin{array}{c} \text{maxRange}=\text{maxDis}-\left(1-\mathrm{pre}\right)\times 0.5\times \text{disLen} \end{array}$$

For each unlabeled LR pair, we compute its distance to the centroid. When its distance does not fall within the computed range, it is taken as a negative sample.

### 2.5 LRI prediction

#### 2.5.1 LRI classification with soft‐margin SVM

Support Vector Machine (SVM) has been popularly applied due to its superior classification accuracy, adequate generalization ability to new samples and without local minima. Inspired by the soft‐margin SVM method proposed by Ref. [23], we explore a novel soft‐margin SVM‐based LRI prediction model, LRI‐sSVM. For an LR pair $x_{i}$, LRI‐sSVM aims to identify a hyperplane defined by Equation (5):

(5) $$\begin{array}{c} \begin{array}{c} \min_{\omega ,\hat{b},\xi_{i}}\;\frac{1}{2}∥\omega {∥}^{2}+\hat{C}\sum_{i=1}^{n}\;\xi_{i}\\ s.t.y_{i}\left(\omega^{T}x_{i}+\hat{b}\right)\geq 1-\xi_{i},\xi_{i}\geq 0,i=1,\ldots ,n \end{array} \end{array}$$

where ω and $\hat{b}$ are two coefficients corresponding to the optimal separating hyperplane, $\xi_{i}$ measures how far an LR pair locates on the wrong side of the hyperplane, $\hat{C}$ weighs model fitting ability and boundary maximization performance.

To acquire a nonlinear separation, each LR pair is mapped to a high‐dimensional space through a projection function $\phi \left(\cdot \right)$. Given a kernel function $K\left(x_{i},x_{s}\right)=\phi \left(x_{i}\right)\cdot \phi \left(x_{s}\right)$, soft‐margin SVM is represented as Equation (6):

(6) $$\begin{array}{c} \begin{array}{c} \max_{\alpha }\;\sum_{i=1}^{n}\;\alpha_{i}-\frac{1}{2}\sum_{i,s=1}^{n}\;\alpha_{i}\alpha_{s}y_{i}y_{s}K\left(x_{i},x_{s}\right)\\ s.t.\sum_{i=1}^{n}\;\alpha_{i}y_{i}=0,0\leq \alpha_{i}\leq \hat{C},i=1,\ldots ,n \end{array} \end{array}$$

The Lagrange multiplier α is computed by Equation (6). Assume that

(7) $$\begin{array}{c} K\left(x_{i},x_{s}\right)={\left(\gamma \left(x_{i},x_{s}\right)+\hat{r}\right)}^{\hat{d}} \end{array}$$

where γ, $\hat{r}$ and $\hat{d}$ denote the coefficient, independent term and the order in the kernel function.

Finally, given an LR pair $x^{*}$ without label, LRI‐sSVM obtains its class by Equation (8):

(8) $$\begin{array}{c} E_{\mathrm{SVM}}=\mathrm{sign}\left(\sum_{i^{\prime }\in \mathrm{SV}}\;\alpha_{i^{\prime }}y_{i^{\prime }}K\left(x_{i^{\prime }},x^{*}\right)+\hat{b}\right) \end{array}$$

where SV is the set of support vectors, i.e. all samples from the training set with $\alpha_{i}>0$.

#### 2.5.2 LRI classification with 1D‐CNN

1D‐CNN exhibits the powerful classification ability and low‐cost hardware implementation. Inspired by 1D‐CNN, we develop 1D‐CNN‐based LRI classification method LRI‐1D‐CNN. LRI‐1D‐CNN contains three CNN layers and two full connection layers.

During training, at each CNN layer, first, given weights and bias of the e‐th filter kernel in the M‐th layer $G_{e}^{M}$ and ${\dot{b}}_{e}^{M}$, we conduct the convolutional operation by Equation (9):

(9) $$\begin{array}{c} \begin{array}{c} k_{e}^{M+1}\left(c\right)=G_{e}^{M}*x^{M}\left(c\right)+{\dot{b}}_{e}^{M} \end{array} \end{array}$$

where $x^{M}\left(c\right)$ represents the c‐th region at the M‐th layer, $k_{e}^{M+1}\left(c\right)$ represents the output of the c‐th neuron related to the e‐th feature map at the $\left(M+1\right)$‐th layer, * represents the dot product operation.

Next, to accelerate the training speed and the model generalization ability, we conduct batch normalization by adding a batch normalization layer after each convolutional layer. The transformation is described by Equation (10):

(10) $$\begin{array}{c} \begin{array}{c} {\hat{k}}^{i}=\frac{k^{i}-E\left[k^{i}\right]}{\sqrt{\mathrm{Var}\left[k^{i}\right]}}\\ J^{i}=\delta^{i}{\hat{k}}^{i}+\eta^{i} \end{array} \end{array}$$

where $E\left[k^{i}\right]$ and $\mathrm{Var}\left[k^{i}\right]$ represent the expectation and variance of the i‐th feature, respectively. $J^{i}$ represents the output of one neuron response. $\delta^{i}$ and $\eta^{i}$ are two parameters to be learned.

Third, an activation function ReLU is utilized to reduce the overfitting problem on all batch normalization layers based on the output $J_{e}^{M+1}\left(c\right)$ by Equation (11):

(11) $$\begin{array}{c} {\tilde{a}}_{e}^{M+1}\left(c\right)=f\left(J_{e}^{M+1}\left(c\right)\right)=\max\left\{0,J_{e}^{M+1}\left(c\right)\right\} \end{array}$$

where ${\tilde{a}}_{e}^{M+1}\left(c\right)$ indicates the output of the o‐th neuron related to the e‐th feature at the $\left(M+1\right)$‐th layer.

Finally, we implement max‐pooling operation with S pool kernels and stride $\hat{j}$ to further prevent overfitting by Equation (12):

(12) $$\begin{array}{c} H_{e}^{M+1}=\max_{\left(\hat{j}-1\right)S+1\leq o\leq \hat{j}S}\;\left\{q_{e}^{M}\left(o\right)\right\} \end{array}$$

where $q_{e}^{M}\left(o\right)$ denotes the output of o‐th neuron related to the e‐th feature map at the M‐th layer and $H_{e}^{M}+1$ is the output of the e‐th feature map at the $\left(M+1\right)$‐th layer. And the full connection layer maps the outputs at the pooling layer to a one‐dimensional vector by Equation (13):

(13) $$\begin{array}{c} \begin{array}{c} E_{1D-\mathrm{CNN}}=D^{M+1}\left(c\right)=f\left(\sum_{e=1}^{N}\;\sum_{o=1}^{I}\;{\tilde{W}}_{\mathrm{eoc}}^{M}{\tilde{a}}_{e}^{M}\left(o\right)+{\tilde{b}}_{c}^{M}\right) \end{array} \end{array}$$

where $f\left(\cdot \right)$ indicates the activation function ReLU, $D^{M+1}\left(c\right)$ indicates the output of the c‐th neuron at the $\left(M+1\right)$‐th layer, and ${\tilde{W}}_{\mathrm{eoc}}^{M}$ is a weight matrix.

During test, for each input LRI $x_{i}$, LRI‐1D‐CNN extracts features through convolution, batch normalization, ReLU and pooling operations, and then computes an association score ($E_{1D-\mathrm{CNN}}$) for each LRI through the full connection layer.

#### 2.5.3 LRI classification with multi‐head attention mechanism

Multi‐head attention (MHA) mechanism plays a significant effect on the model performance. Differed from single‐head attention mechanism, MHA module splits the full hidden space to several parallel subspaces. Hence, we design an MHA‐based LRI prediction model LRI‐MHA. First, we normalize LRIs with batch normalization by Equation (10) and obtain the normalized data $\tilde{X}$. Next, we extract LRI features through MHA. Through linear transformation of $\tilde{X}$, we can obtain $\text{Query}\;\left(Q\right)$, $\mathrm{Key}\;\left(K\right)$ and $\text{Value}\;\left(V\right)$ by Equations ((14), (15), (16)):

(14) $$\begin{array}{c} Q=\text{Linea}r_{1}\left(\tilde{X}\right)=W^{q}\tilde{X} \end{array}$$

(15) $$\begin{array}{c} K=\text{Linea}r_{2}\left(\tilde{X}\right)=W^{k}\tilde{X} \end{array}$$

(16) $$\begin{array}{c} V=\text{Linea}r_{3}\left(\tilde{X}\right)=W^{v}\tilde{X} \end{array}$$

The output of a single‐head attention is represented as Equation (17):

(17) $$\begin{array}{c} \text{Attention}\left(Q,K,V\right)=\text{Softmax}\left(\frac{QK^{T}}{\sqrt{d_{k}}}\right)V \end{array}$$

By stacking h times parallel scaled dot‐product attention, the MHA module is described by Equations (18) and (19):

(18) $$\begin{array}{c} \mathrm{hea}d_{h}=\text{Attention}\left(QW_{h}^{q},KW_{h}^{k},VW_{h}^{v}\right),h=1,2,\ldots ,8 \end{array}$$

(19) $$\begin{array}{c} F=\text{MultiHead}\left(Q,K,V\right)=\text{Concat}\left(\mathrm{hea}d_{1},\ldots ,\mathrm{hea}d_{h}\right)W^{o} \end{array}$$

where $W_{h}^{q}\in {\mathbb{R}}^{d_{\text{model}}\times d_{q}}$, $W_{h}^{k}\in {\mathbb{R}}^{d_{\text{model}}\times d_{k}}$, $W_{h}^{v}\in {\mathbb{R}}^{d_{\text{model}}\times d_{v}}$, $W^{o}\in {\mathbb{R}}^{hd_{v}\times d_{\text{model}}}$, $d_{q}=d_{k}=d_{v}=d_{\text{model}}/h=50$.

The output at the MHA layer is represented by Equation (20):

(20) $$\begin{array}{c} E_{\mathrm{MHA}}=\text{ReLU}\left(\tilde{W}F+\tilde{b}\right) \end{array}$$

where $E_{\mathrm{MHA}}$ can be used as LRI association score matrix.

#### 2.5.4 Ensemble learning

Ensemble learning typically exhibits superior learning outcomes compared to single classifiers. Hence, we design a Stacking‐based heterogeneous ensemble learning model to fuse a plethora of distinct machine learning algorithms. We first train three basic models (i.e. SVM, 1D‐CNN and MHA) and then train meta‐model to generate meta‐features.

First, the training set is bifurcated to a basic training set which contains 70% samples and a meta‐training set which contains 30% samples. The basic training set is used to independently train SVM, 1D‐CNN and MHA. Each model among three models yields the predictions corresponding to each sample, each of which contains two scores, thereby generating six meta‐features by Equation (21).

(21) $$\begin{array}{c} \begin{array}{c} E=[E_{SVM\left(y_{i}=0\right)},E_{SVM\left(y_{i}=1\right)},E_{1D‐CNN\left(y_{i}=0\right)},\\ \;E_{1D‐CNN\left(y_{i}=1\right)},E_{MHA\left(y_{i}=0\right)},E_{MHA\left(y_{i}=1\right)}] \end{array} \end{array}$$

Next, the obtained meta‐features E are fed to a multilayer perceptron with three full connection layers for predicting the interaction probability $P_{\text{Meta}}$ of each LRI by Equation (22):

(22) $$\begin{array}{c} P_{\text{Meta}}=\text{Softmax}\left(\tilde{W}E+\tilde{b}\right) \end{array}$$

For the i‐th LRI, if $P_{\text{Meta}\left(y_{i}=1\right)}$ is larger than $P_{\text{Meta}\left(y_{i}=0\right)}$, the LRI is taken as interacting, otherwise, it is taken as no‐interacting.

During the training process, we employ cross‐entropy loss to quantify the discrepancy between predicted and actual outcomes by Equation (23):

(23) $$\begin{array}{c} \text{Loss}\left({\hat{P}}_{i},P_{i}\right)=-\frac{1}{n}\sum_{i=1}^{n}\;\left[P_{i}\log\left({\hat{P}}_{i}\right)-\left(1-P_{i}\right)\log\left(1-{\hat{P}}_{i}\right)\right] \end{array}$$

where ${\hat{P}}_{i}$ and $P_{i}$ represent the probabilities of the true label and the predicted label, respectively.

To further minimize the loss, we use the gradient descent method in the Adam optimizer to fine‐tune the parameters based on the Cosine Annealing learning rate scheduler by Equations ((24), (25), (26), (27)):

(24) $$\dot{m_{t}}\begin{array}{c} =\beta_{1}{\dot{m}}_{t-1}+\left(1-\beta_{1}\right)\dot{g_{t}} \end{array}$$

(25) $$\begin{array}{c} \dot{v_{t}}=\beta_{2}{\dot{v}}_{t-1}+\left(1-\beta_{2}\right){{\dot{g}}_{t}}^{2} \end{array}$$

(26) $$\begin{array}{c} \text{variable}=\text{variable}-lr_{t}\times \frac{\dot{m_{t}}}{\sqrt{\dot{v_{t}}}+\varepsilon } \end{array}$$

(27) $$\begin{array}{c} \begin{array}{c} lr_{t}=lr_{\min}+\frac{1}{2}\left(lr_{\max}-lr_{\min}\right)\left(1+\cos\left(\frac{T_{\mathrm{cur}}}{T_{\max}}\times \pi \right)\right) \end{array} \end{array}$$

where $\dot{m_{t}}$ and $\dot{v_{t}}$ represent the first‐order and second‐order moments of the gradient $\dot{g_{t}}$, respectively, $\beta_{1}$ and $\beta_{2}$ indicate corresponding exponential decay rates, respectively. variable and $lr_{t}$ denote the parameter and the learning rate, and ε represents a small constant for avoiding division by zero. $lr_{\min}$ and $lr_{\max}$ represent the minimum and maximum learning rates, respectively. $T_{\mathrm{cur}}$ and $T_{\max}$ indicate the current and maximum iteration numbers, respectively.

### 2.6 CCC inference

In Ref. [4] Peng et al. developed a three‐point evaluation approach for CCC scoring and obtained better performance. In this study, we first conduct LRI filtering and then evaluate CCC scores using the three‐point evaluation approach. The three‐point evaluation approach, which comprises cell expression, expression product and specific expression, can reduce the influence caused by individual models on the performance.

#### 2.6.1 LRI filtering

ScRNA‐seq data offers a wealth of expression data for ligands and receptors, aiding in the construction of cellular communication network. To assess the CCC intensity, we first conduct filtering for the predicted LRIs through a threshold θ to preserve only LRIs with interaction probabilities greater than θ. Next, we download melanoma scRNA‐seq data from the GEO database,^24^ and further conduct filtering known and predicted LRIs. If an LRI is not expressed within these cells, it is not considered to mediate the CCC.

#### 2.6.2 LRI_*u,w,j,p*_ computation

##### The cell expression approach

The cell expression approach initially calculates the quantity of cells, ${\hat{c}}_{u,j}$ and ${\hat{c}}_{w,p}$, where the ligand u and receptor w are expressed in $C_{j}$ and $C_{p}$ respectively. Next, the interaction score between ligand u and receptor w that mediate communication between $C_{j}$ and $C_{p}$ is quantified by Equation (28):

(28) $$\begin{array}{c} \begin{array}{c} \mathrm{LR}I_{u,w,j,p}^{1}=\frac{{\hat{c}}_{u,j}}{{\hat{n}}_{1}}\times \frac{{\hat{c}}_{w,p}}{{\hat{n}}_{2}} \end{array} \end{array}$$

where ${\hat{n}}_{1}$ and ${\hat{n}}_{2}$ represent the total number of cells in $C_{j}$ and $C_{p}$ respectively.

##### The expression product approach

The expression product approach calculates the interaction score between ligand u and receptor w that mediate communication between $C_{j}$ and $C_{p}$ by Equation (29):

(29) $$\begin{array}{c} \begin{array}{c} \mathrm{LR}I_{u,w,j,p}^{2}={\bar{L}}_{u,j}\times {\bar{R}}_{w,p} \end{array} \end{array}$$

##### The specific expression approach

The specific expression approach calculates the interaction score between ligand u and receptor w that mediate communication between $C_{j}$ and $C_{p}$ by Equation (30):

(30) $$\begin{array}{c} \mathrm{LR}I_{u,w,j,p}^{3}=\frac{{\bar{L}}_{u,j}}{\sum_{j=1}^{m}\;{\bar{L}}_{u,j}}\times \frac{{\bar{R}}_{w,p}}{\sum_{p=1}^{m}\;{\bar{R}}_{w,p}} \end{array}$$

#### 2.6.3 CCC strength measurement

For m cell types $\left\{C_{1},C_{2},\ldots ,C_{m}\right\}$ and v filtered LRIs $\left\{\left(l_{1}r_{1}\right),\left(l_{2},r_{2}\right),\ldots ,\left(l_{v},r_{v}\right)\right\}$, we use a three‐point evaluation approach to calculate CCC strength by combining the above three methods. The CCC score $f_{1}\left(j,p\right)$ from $C_{j}$ to $C_{p}$ based the cell expression approach is calculated by Equation (31):

(31) $$\begin{array}{c} f_{1}\left(j,p\right)=\sum_{u,w=1}^{v}\;\mathrm{LR}I_{u,w,j,p}^{1} \end{array}$$

Similarly, the CCC scores $f_{2}\left(j,p\right)$ and $f_{3}\left(j,p\right)$ from $C_{j}$ to $C_{p}$ can be calculated by Equations (29) and (30), respectively.

Subsequently, the min‐max scaling method is used to normalize $f_{1}\left(j,p\right)$, $f_{2}\left(j,p\right)$ and $f_{3}\left(j,p\right)$ and achieve the normalized CCC scores $g_{1}$, $g_{2}$ and $g_{3}$, respectively. Assume that the maximum value, minimum value and median value among $g_{1}$, $g_{2}$ and $g_{3}$ are represented as $g_{\max}$, $g_{\min}$ and $g_{\mathrm{med}}$, respectively. Finally, the CCC score from $C_{j}$ to $C_{p}$ is calculated with the three‐point estimation approach by Equation (32):

(32) $$\begin{array}{c} f\left(j,p\right)=\frac{g_{\max}+4\times g_{\mathrm{med}}+g_{\min}}{6} \end{array}$$

## 3 RESULTS

### 3.1 Experimental settings and evaluation metrics

5‐fold cross‐validation is a commonly‐used technique for training and evaluating models. In this process, a dataset is divided into five equal parts, where four parts are used for training the model and the remaining is used for testing. In this study, to gauge the capabilities of SEnSCA in the LRI classification, we repeatedly executed 5‐fold cross validation for 20 times, with each dataset being shuffled to ensure that each part was used as a test set at each time. Due to the substantial volume of unlabeled LRIs, we set pre to 0.6 in the K‐means algorithm, thereby eliminating half of the unlabeled samples to better adapt to the requirements.

For the SVM model, optimal parameters were determined through grid search. A parameter grid was defined based on various combinations of ‘C', ‘gamma’ and ‘kernel’: ‘C': [1.5, 2.5, 3.5], ‘gamma’: [0.1, 0.5, 1], ‘kernel’: [‘poly’, ‘rbf’, ‘sigmoid’]. Following grid search and cross‐validation, the optimal parameter combination was identified as C = 2.5, gamma = 0.1, kernel = ‘poly’. The 1D‐CNN model comprised 3 convolutional layers, 3 batch normalization layers, 3 max‐pooling layers and 2 fully connection layers. The MHA included 1 batch normalization layer, 1 dropout layer and 2 fully connection layers. The meta‐classifier was a three‐layer fully connection network. The initial parameters and configurations for LRI‐sSVM, LRI‐1D‐CNN, LRI‐MHA and Meta‐classifier models were shown in Tables 1 and 2.

**TABLE 1:** Parameter settings.

Method	Parameter settings
SVM	Gamma = 0.1, C = 2.5, kernel=’poly’, tol = 0.001, Cache_size = 100, probability = True
1D‐CNN	Num_epochs = 30, lr = 0.001, Weight_decay = 1e^−5^, batch_size = 64
MHA	Input_dim = 400, num_classes = 2, Num_heads = 8, hidden_size = 256
Meta‐classifier	Optimizer = Adam (lr = 0.01), Scheduler = CosineAnnealingLR (eta_min = 1e^−6^)

**TABLE 2:** Configuration of SEnSCA.

Method	Layer	Configuration
1D‐CNN	Convolution layer 1 Batch normalization layer 1 Max‐pooling layer 1 Convolution layer 2 Batch normalization layer 2 Max‐pooling layer 2 Convolution layer 3 Batch normalization layer 3 Max‐pooling layer 3 Fully connection layer 1 Fully connection layer 2	32 filters, 3 × 1 kernel and 1 stride 32 n_features and ReLU 2 × 1 kernel, 2 stride 64 filters, 3 × 1 kernel and 1 stride 64 n_features and ReLU 2 × 1 kernel, 2 stride 128 filters, 3 × 1 kernel and 1 stride 128 n_features and ReLU 2 × 1 kernel, 2 stride 128 neurons, ReLU 2 neurons
MHA	Batch normalization layer Fully connection layer 1 Dropout layer Fully connection layer 2	Input_dim = 400 256 neurons, ReLU 20% 2 neurons
Meta‐classifier	Fully connected layer 1 Fully connected layer 2 Fully connected layer 3	12 neurons, ReLU 8 neurons, ReLU 2 neurons, softmax

LRI prediction can be taken as a binary classification task. By using multiple evaluation metrics, we can more fairly and objectively represent the SEnSCA performance. In this study, we used AUC (area under the ROC curve), AUPR (area under the precision‐recall curve), precision, recall, accuracy, F1‐score and the Jaccard index as evaluation metrics. For AUC, abscissa and ordinate corresponding to its ROC curve were False Positive Rate (FPR) and True Positive Rate (TPR), respectively. For AUPR, abscissa and ordinate corresponding to its PR curve is recall and precision, respectively.

(33) $$\begin{array}{c} \mathrm{FPR}=\frac{\mathrm{FP}}{\mathrm{FP}+\mathrm{TN}} \end{array}$$

(34) $$\begin{array}{c} \mathrm{TPR}=\frac{\mathrm{TP}}{\mathrm{TP}+\mathrm{FN}} \end{array}$$

(35) $$\begin{array}{c} \text{Precision}=\frac{\mathrm{TP}}{\mathrm{TP}+\mathrm{FP}} \end{array}$$

(36) $$\begin{array}{c} \text{Recall}=\frac{\mathrm{TP}}{\mathrm{TP}+\mathrm{FN}} \end{array}$$

(37) $$\begin{array}{c} \text{Accuracy}=\frac{\mathrm{TP}+\mathrm{TN}}{\mathrm{TP}+\mathrm{FP}+\mathrm{TN}+\mathrm{FN}} \end{array}$$

(38) $$\begin{array}{c} F1-\text{score}=\frac{2\times \text{Precision}\times \text{Recall}}{\text{Precision}+\text{Recall}} \end{array}$$

The Jaccard index was used to evaluate the similarity between two sets.

(39) $$\overset{˙}{J\;}\;\begin{array}{c} \left(O_{\hat{p}},O_{\hat{q}}\right)=\frac{\left\|\left(O_{\hat{p}}\cap O_{\hat{q}}\right)\right\|}{\left\|\left(O_{\hat{p}}\cup O_{\hat{q}}\right)\right\|} \end{array}$$

where $O_{\hat{p}}$ and $O_{\hat{q}}$ denotes LRIs provided by two different CCC inference tools, respectively. $O_{\hat{p}}\cap O_{\hat{q}}$ and $O_{\hat{p}}\cup O_{\hat{q}}$ denote their intersection and union, respectively. $\left\|\cdot \right\|$ represents the number of elements in a set.

### 3.2 Comparison of SEnSCA with other methods

In this section, we conducted a comparative analysis between SEnSCA and five methods: PIPR,^25^ XGBoost,^26^ DNNXGB,^27^ OR‐RCNN,^28^ and CellComNet.^6^

PIPR used Siamese residual and protein sequences for PPI prediction. XGBoost is a novel PPI sites prediction method through XGBoost. DNNXGB used deep neural network and XGBoost for predicting PPIs. OR‐RCNN computed confidence score for each PPI via ordinal regression and recurrent convolutional neural network. CellComNet used heterogeneous Newton boosting machine for LRI classification and a joint scoring strategy for CCC scoring.

**FIGURE 2:**
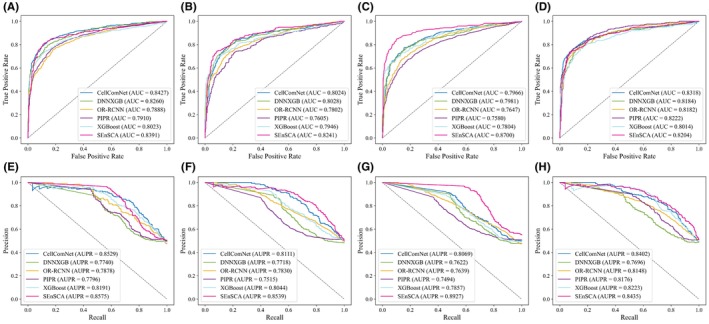
The ROC and PR curves obtained by six LRI prediction models on four datasets. (A–D) denote their ROC curves on the four datasets, respectively. (E–H) denote their PR curves, respectively.

Figure 2 provides a comprehensive demonstration of the ROC and PR curves of the above six LRI inference models on four distinct datasets. Based on the results, we observed that for SEnSCA, on Dataset 1, its AUC was slightly lower than that of CellComNet, but still higher than the other four methods. More importantly, its AUPR exceeded all five methods. Compared to CellComNet, SEnSCA's lower AUC could be due to differences in model architecture, feature representation and data distribution.

On Datasets 2 and 3, its performance significantly outperformed the other five models. On Dataset 4, its performance was slightly inferior to CellComNet, but it still surpassed the other four methods. In summary, on the four datasets, its average AUC was 0.8384, outperforming 2%, 2.71%, 5.04%, 5.55% and 4.37% compared to the other five methods, respectively. And its average AUPR was 0.8619 with higher than the other methods by 3.41%, 9.25%, 7.45%, 8.74% and 5.4%, respectively. That is, SEnSCA significantly improved LRI classification performance in most cases, reflecting its strong LRI prediction capability.

### 3.3 Performance comparison for negative LRI sample construction

On four LRI datasets, there are only a small number of positive LRI samples and a large number of unlabeled samples, but there is no negative LRI samples. To assess the effect of the selected negative LRIs on the classification performance, we randomly selected negative LRIs from unlabeled ligand‐receptor pairs and combined with known LRIs, then ran the classifier. At the same time, we still adopted a K‐means‐based method to select negative LRIs and combined with positive LRIs for LRI identification.

**FIGURE 3:**
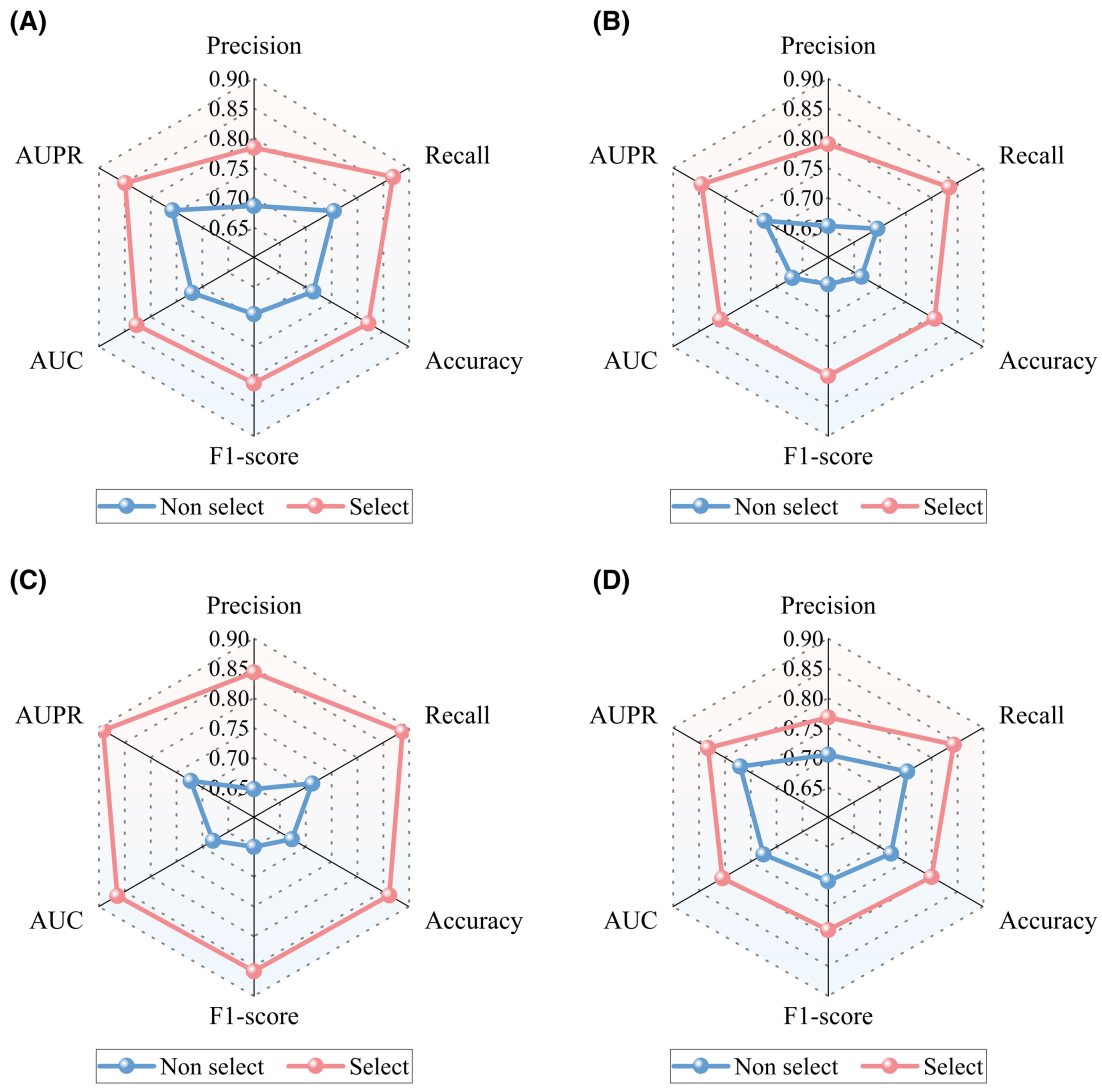
Performance comparison of two negative LRI selection methods: "Non select" and "Select".

To compare the performance of the above two methods, we used six evaluation metrics, namely precision, recall, accuracy, F1‐score, AUC and AUPR. Through comparative experiments, as shown in Figure 3, the K‐means‐based negative LRI selection method obtained better performance than the random selection method on the four datasets. This finding further emphasized the effectiveness and superiority of the K‐means approach in negative sample selection, providing an effective strategy for handling similar issues.

### 3.4 Comparison within three tumour tissues

**FIGURE 4:**
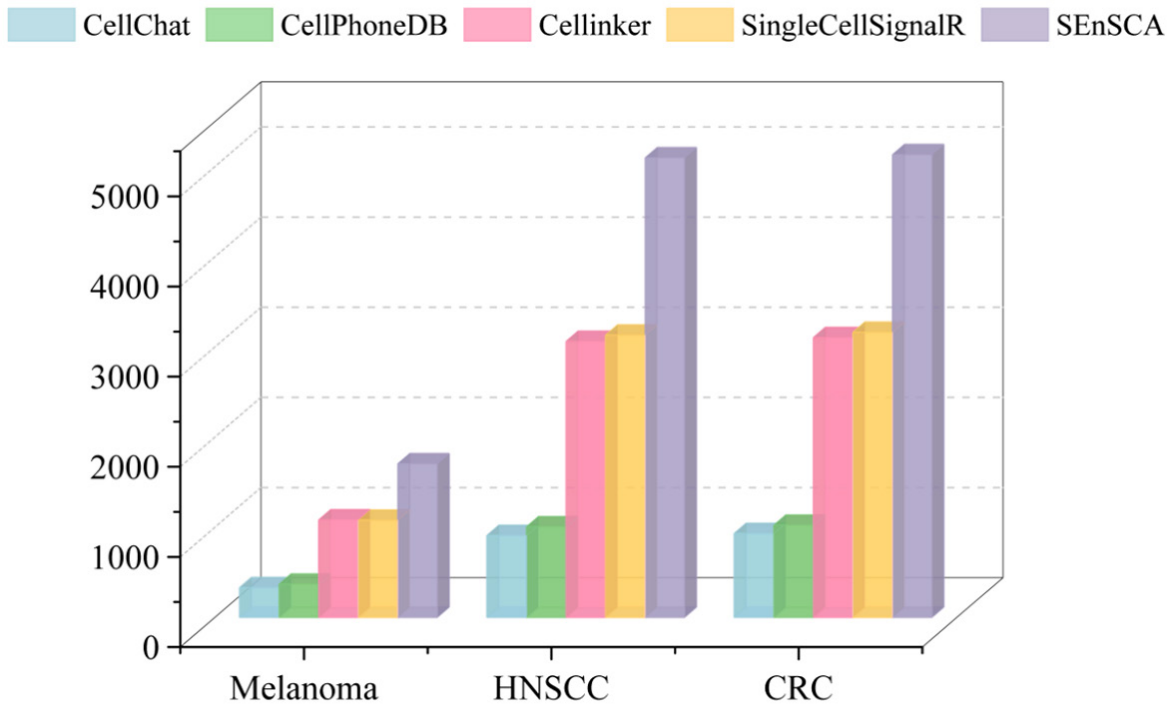
The number of the filtered LRI provided by CellChat, CellPhoneDB, Cellinker, SingleCellSignalR and SEnSCA within melanoma, HNSCC, and CRC.

To evaluate the SEnSCA performance, we conducted a comparative analysis with four cutting‐edge CCC inference analysis tools: CellChat,^29^ CellPhoneDB,^12^ Cellinker^10^ and SingleCellSignalR.^30^ We focused on three tumour tissues: melanoma (accession code: GSE72056), head and neck squamous cell carcinomas (HNSCC) (accession code: GSE103322) and colorectal cancer (CRC) (accession code: GSE81861). Leveraging scRNA‐seq data from GEO,^24^ we conducted filtering for all LRIs obtained by these methods. Figure 4 gives the number of LRIs after filtering.

**FIGURE 5:**
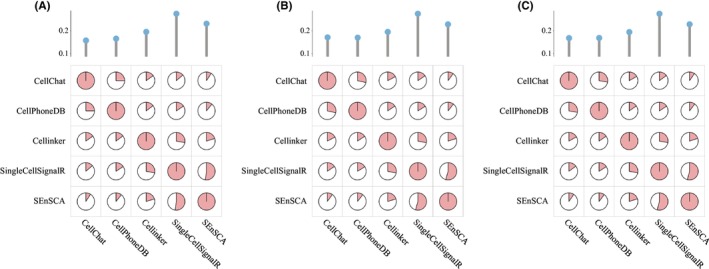
Jaccard index between any two methods after filtering LRIs in three cancer tissues. The average Jaccard index is displayed at the top of the pie plot. (A) Melanoma (B) CRC (C) HNSCC.

In addition, we also calculated the Jaccard index of SEnSCA and four CCC tools, that is, CellChat,^29^ CellPhoneDB,^12^ Cellinker^10^ and SingleCellSignalR^30^ within the three tissues. Figure 5 gives their Jaccard index. The average Jaccard index between a tool and the remaining is depicted using a lollipop plot. Notably, SEnSCA computed the second‐best Jaccard index within the three tissues, higher than that of CellChat, CellPhoneDB and Cellinker. SEnSCA calculated a smaller Jaccard value than SingleCellSignalR with lower than 0.0430, 0.0479 and 0.0472, respectively. This may be due to that SEnSCA acquired a large number of new LRIs. In the case of similar numerators, the larger the denominator is, the lower the Jaccard index is.

### 3.5 LRI validation by molecular docking

To validate the predicted LRIs by SEnSCA, we conducted molecular docking. Molecular docking is a widely‐used computational chemistry tool for measuring molecular interactions. It assumes that a lower molecular binding energy between an LRI indicates a higher interaction likelihood. In this study, we randomly selected 30 LRIs from the predicted LRIs for molecular docking on each dataset.

To conduct molecular docking, we used an online tool ZDOCK^31^ and obtained structures of both ligand and receptor from an LRI. Next, molecular docking between the ligand and receptor was conducted with PDBePISA^32^ with default parameters. And interface area (IA, Å^2^), binding energy (BE, kcal/mol), number of hydrogen bonds (N_HB_), number of salt bridges (N_SB_), hydrogen bond (HB, Å) and salt bridge (SB, Å) were calculated. LRI with binding energy lower than −4 kcal/mol was considered potential LRI.

Table 3 presents the molecular docking information of the LRI with the smallest binding energy in each dataset. The results indicated that all four LRIs exhibited obviously low binding energy, suggesting a strong interaction likelihood. For a comprehensive view, the detailed molecular docking for the 30 selected LRIs in each dataset can be downloaded at https://github.com/plhhnu/SEnSCA/Validation/Docking. These results contribute to our understanding of potential LRIs and provide valuable insights into cellular communication dynamics.

**TABLE 3:** The molecular docking results of LRIs with the smallest BE on four datasets.

Dataset	Ligand	Receptor	Interface area	BE	N_HB_	N_SB_	Hydrogen bonds	Salt bridges
Dataset 1	APOC1	ICOS	5899.3	−92.3	49	11	2.77	3.23
Dataset 2	Avp	Icos	2808.3	−45.1	14	6	3.15	3.77
Dataset 3	Pgf	Pvr	3424.3	−56.9	19	0	3.32	0
Dataset 4	CGA	MYDGF	3954.5	−50.4	49	3	2.97	2.34

### 3.6 Comparison of SEnSCA and other four CCC analysis tools

We quantified the overlapping LRIs between SEnSCA and other four CCC analysis tools, namely, CellChat,^29^ Connectome,^33^ CytoTalk,^34^ and NATMI.^35^ In SEnSCA, LRIs with interaction probability greater than 0.99 were designated as potential LRIs. Thus, SEnSCA identified 1938, 1640, 1875 and 7003 novel LRIs on the four datasets, respectively. By combining known and predicted LRIs, an aggregate of 5328, 3671, 3881 and 13,588 LRIs were obtained, respectively. Next, we observed the overlapping LRIs between SEnSCA and the above four databases. Table 4 enumerates the number of the overlapping LRIs and the Jaccard index between SEnSCA and the four databases, in conjunction with the total overlapping LRI number and the Jaccard index, respectively. After discarding repeated LRIs, there were 2495 overlapping LRIs between SEnSCA and the four tools on Dataset 1. Moreover, Venn diagrams, where the intersection part epitomizes elements shared by multiple sets and the independent part signifies elements that solely exist in an individual set, were employed to portray overlaps and relationships among different sets. For a more comprehensive understanding of the overlapping LRIs between SEnSCA and the other four tools, detailed information can be found in the Venn diagrams at https://github.com/plhhnu/SEnSCA/Venn. The results demonstrated that SEnSCA captured an abundance of overlapping LRIs with CellChat, Connectome, CytoTalk and NATMI, thus verifying the reliability of the identified LRIs.

**TABLE 4:** The overlapping LRI number and the Jaccard index between SEnSCA and four CCC databases.

Dataset	CellChat	Connectome	CytoTalk	NATMI	Total
Dataset 1	567/8.5%	2323/41.8%	1788/32.6%	1866/32.4%	2495/34.3%
Dataset 2	484/9.3%	1262/25.4%	1146/26.2%	/	1343/20.3%
Dataset 3	437/8.0%	1983/44.5%	1533/36.5%	/	2000/32.5%
Dataset 4	164/1.1%	455/2.9%	433/2.9%	440/2.8%	490/2.8%

### 3.7 Ablation study

**FIGURE 6:**
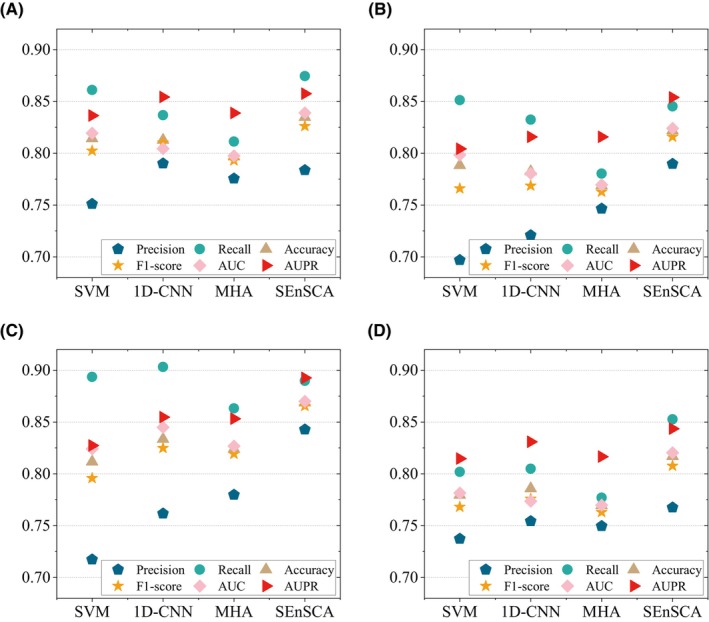
The performance of SEnSCA and three individual models (SVM, 1D‐CNN and MHA) on four LRI datasets.

SEnSCA is an ensemble model comprising LRI‐sSVM, LRI‐1D‐CNN and LRI‐MHA. To assess the impact of Stacking ensemble on the LRI prediction performance, we conducted a detailed comparison between SEnSCA and its basic models. As shown in Figure 6, across the four LRI datasets, the SEnSCA performance surpassed LRI‐sSVM, LRI‐1D‐CNN and LRI‐MHA, achieving higher scores in accuracy, F1‐score, AUC and AUPR. These results strongly demonstrated that ensemble learning can enhance the LRI classification performance, enabling SEnSCA to effectively identify reliable LRIs.

### 3.8 CCC inference within human melanoma tissues

Heterogeneity of tumours is one of the main features of malignant tumours.^36^, ^37^, ^38^ Melanoma is a malignant skin tumour caused by extreme proliferation of abnormal melanocytes. It has the highest metastasis and recurrence rate among skin cancers.^39^ Its rapid growth and metastatic ability make its early diagnosis and treatment pivotal. By studying its CCC changes, we can better understand its development mechanism and provide more precise diagnoses and personalized treatment strategies for patients.

To analyse melanoma ligand‐receptor co‐expression patterns, we analysed its transcriptomes comprising about 4000 cells provided by Ref. [40] in seven cell types in its single‐cell suspensions: melanoma cancer cells, cancer‐associated fibroblasts (CAFs), macrophages, endothelial cells, T cells, B cells and natural Killer cells. We obtained its scRNA‐seq data from the GEO database (accession code: GSE72056).^24^ For an LRI, if its ligand or receptor is not expressed in the corresponding cells, we do not consider it as a valid communication medium. Thus, in Dataset 1, we identified 1707 LRIs related to melanoma. Table 5 lists the top three LRIs mediating communication between melanoma cancer cells and other cell types based on the three CCC scoring methods, and the top three LRIs mediating communication between melanoma cells and other cell types.

**TABLE 5:** Intercellular communication prediction results based on different scoring approaches in human melanoma tissues.

Function	Cell type	“outgoing” LRIs	“incoming” LRIs
Cell	CAFs Macrophages ECs T cells NK cells B cells	PSAP_LRP1, PTMA_THY1, HSP90B1_LRP1 LGALS1_PTPRC, HLA‐A_APLP2, PTMA_CD53 HLA‐A_APLP2, B2M_HLA‐F, PTMA_TNFRSF9 LGALS1_PTPRC, PTMA_CD3G, B2M_CD3G PTMA_KLRK1, LGALS1_PTPRC, B2M_KLRK1 PTMA_CD79A, B2M_CD79A, LGALS1_PTPRC	CXCL12_RPSA, PSAP_SORT1, APP_RPSA PSAP_SORT1, HLA‐A_APLP2, GRN_SORT1 APP_RPSA, PSAP_SORT1, HLA‐A_APLP2 HLA‐A_APLP2, B2M_HLA‐F, PTMA_C1QBP HLA‐A_APLP2, B2M_HLA‐F, PTMA_C1QBP HLA‐A_APLP2, B2M_HLA‐F, PTMA_C1QBP
Product	CAFs Macrophages ECs T cells NK cells B cells	B2M_THY1, PTMA_THY1, S100A10_THY1 PTMA_CD53, LGALS1_PTPRC, HLA‐A_APLP2 B2M_HLA‐F, HLA‐A_APLP2, CALM2_AQP1 LGALS1_PTPRC, B2M_HLA‐F, B2M_CD3G B2M_KLRK1, LGALS1_KLRK1, B2M_CD247 B2M_CD79A, PTMA_CD79A, S100A10_CD79A	CXCL12_RPSA, B2M_HLA‐F, APP_RPSA B2M_HLA‐F, APOC1_RPSA, HLA‐A_APLP2 APP_RPSA, B2M_HLA‐F, HLA‐A_APLP2 B2M_HLA‐F, HLA‐A_APLP2, PTMA_C1QBP B2M_HLA‐F, HLA‐A_APLP2, PTMA_C1QBP B2M_HLA‐F, PTMA_C1QBP, HLA‐A_APLP2
Specific	CAFs Macrophages ECs T cells NK cells B cells	DLL3_NOTCH3, LIPH_LPAR1, UCN2_THY1 SAA1_FPR1, SAA1_FPR2, CAMP_FPR2 UCN2_GPIHBP1, FGF4_FGFR3, LPL_GPIHBP1 UCN2_ICOS, S100A1_ICOS, GAST_ICOS UCN2_KLRK1, IL9_IL9R, PTH_KLRB1 UCN2_CD79A, OBP2A_OR1G1, PTH_KLRF2	WNT2_FZD9, WNT11_KLRG2, CCL26_BAMBI C1QA_CSPG4, DEFB1_BAMBI, CXCL11_BAMBI AMH_AMHR2, TGFA_ERBB3, VWF_ITGB3 WNT7A_FZD9, WNT1_FZD9, INS_BAMBI AREG_ERBB3, NCAM1_ROBO1, NODAL_ACVR2B RLN3_RXFP3, COL4A3_ITGA3, COL4A3_ITGB3
SEnSCA	CAFs Macrophages ECs T cells NK cells B cells	B2M_THY1, SERPINE2_LRP1, PTMA_THY1 LGALS1_PTPRC, PTMA_CD53, HLA‐A_APLP2 B2M_HLA‐F, HLA‐A_APLP2, GRN_TNFRSF1A LGALS1_PTPRC, B2M_CD3G, B2M_HLA‐F LGALS1_KLRK1, B2M_KLRK1, LGALS1_PTPRC B2M_CD79A, PTMA_CD79A, S100A10_CD79A	CXCL12_RPSA, COL1A1_DDR1, B2M_HLA‐F C1QA_CSPG4, B2M_HLA‐F, PSAP_SORT1 APP_RPSA, B2M_HLA‐F, VWF_ITGB3 B2M_HLA‐F, HLA‐A_APLP2, PTMA_C1QBP B2M_HLA‐F, HLA‐A_APLP2, PTMA_C1QBP B2M_HLA‐F, HLA‐A_APLP2, PTMA_C1QBP

**FIGURE 7:**
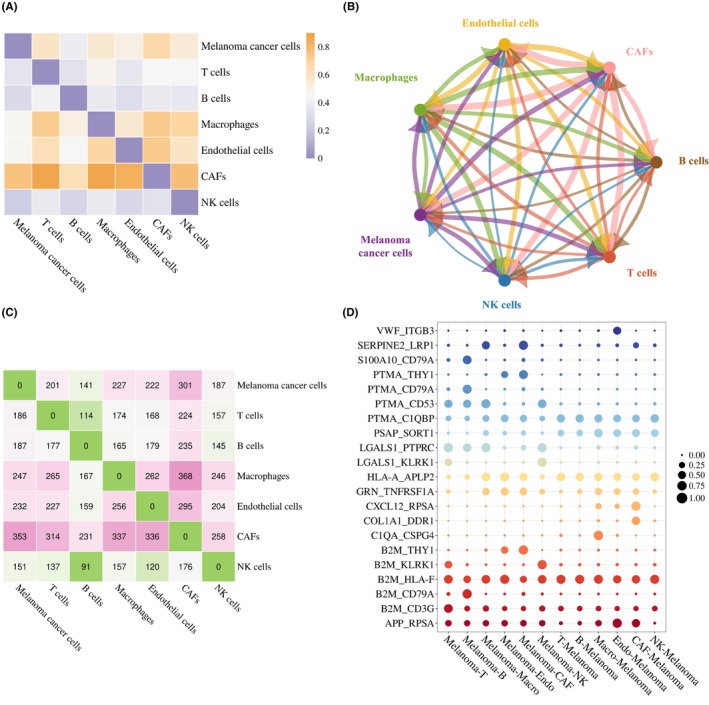
CCC inference results of SEnSCA within melanoma tissues. (A) CCC strength. (B) The CCC network. (C) The number of LRIs mediating corresponding CCC. (D) The top 3 LRIs mediating corresponding CCC.

As shown in Figure 7, we employed three visualization tools (i.e. heatmap, bubble diagram and network diagram) to visualize human melanoma CCC network. Figure 7A (heatmap) shows the intensity of communication between different cell types, with closer to orange indicating greater communication. Figure 7B (network diagram) shows the intensity of communication between different cell types, with thicker lines indicating greater communication. Figure 7C (heatmap) shows the number of LRIs that correspond to CCC between different cell types. Figure 7D (bubble diagram) shows the expression of LRIs between different cell types, where larger bubbles mean that LRIs were more likely to mediate the corresponding CCC.

To evaluate the SEnSCA performance in inferring CCC, as shown in Table 6, we compared its results with those of iTALK,^41^ CellPhoneDB,^12^ NATMI,^35^ CellComNet,^6^ CellDialog.^4^ All methods demonstrated strong CCC inference capabilities and yielded similar results in human melanoma tissue, that is, CAFs and macrophages were more likely to communicate with melanoma cells. Recent findings have underscored the role of CAFs in the progression, metastasis as well as drug resistance of melanoma.^42^ Meantime, the recruitment of macrophages obviously promotes the spread of melanoma cells. Macrophages inhibit melanoma by limiting tumour‐derived vesicle‐B cell communication.^43^

**TABLE 6:** Comparison of SEnSCA with iTALK, CellPhoneDB, NATMI, CellComNet and CellDialog in melanoma.

Ranking	SEnSCA_cell_	SEnSCA_pro_	SEnSCA_spe_	SEnSCA	iTALK	CellPhoneDB	NATMI	CellComNet	CellDialog
1	CAFs	CAFs	CAFs	CAFs	CAFs	Macrophages	CAFs	CAFs	CAFs
2	Macrophages	Macrophages	T cells	Macrophages	Macrophages	ECs	ECs	Macrophages	Macrophages
3	ECs	NK cells	ECs	ECs	ECs	CAFs	T cells	ECs	ECs
4	T cells	ECs	Macrophages	T cells	NK cells	T cells	Macrophages	NK cells	NK cells
5	NK cells	T cells	B cells	NK cells	T cells	B cells	B cells	T cells	T cells
6	B cells	B cells	NK cells	B cells	B cells	NK cells	NK cells	B cells	B cells

Abbreviation: ECs, endothelial cells.

## 4 DISCUSSION

CCC has a crucial role in tumour progression, metastasis and therapeutic resistance. Inferring CCC provides valuable insights into disease mechanisms and facilitates the discovery of novel treatment strategies. The inference of CCC involves two main procedures: acquiring high‐confidence LRIs and measuring CCC strength.

In this work, we proposed SEnSCA to predict LRIs and conduct CCC inference. During LRI prediction, first, we performed feature extraction for each LRI using the iFeature platform based on protein sequences. Next, principal component analysis was applied to perform dimensionality reduction. Third, negative LRI samples were constructed through a K‐means clustering. Finally, new LRIs were identified by a Stacking ensemble model comprising three primary learners (i.e. SVM, 1D‐CNN and MHA). Following LRI prediction, CCC strength was computed by integrating LRI filtering and scRNA‐seq data. More importantly, we visualized the constructed CCC network.

We conducted a series of experiments for evaluating the SEnSCA performance: first, it was compared with four cutting‐edge PPI identification models, namely, DNNXGB, OR‐RCNN, PIPR and XGBoost, along with the latest LRI prediction method, CellComNet. Second, it was compared with four classical CCC analysis tools, that is, CellChat, Connectome, CytoTalk and NATMI across all four LRI datasets. It not only had a greater number of overlapping LRIs but also demonstrated a high Jaccard index with each of the four databases. Third, it randomly selected 30 predicted LRIs and conducted molecular docking experiments on each LRI dataset. The molecular binding energies of the 30 LRIs were remarkably low, further attesting their high confidence. Finally, it was pitted against other four CCC inference tools (i.e. CellChat, CellPhoneDB, iTALK and NATMI) within human melanoma tissues. As a result, it obtained better CCC analysis results, which were basically consistent with that of the four tools.

The main advantages of SEnSCA contain the following points: (i) it extracted rich LRI features from protein sequences; (ii) it utilized a K‐means‐based method to construct negative samples, ensuring a balance between positive and negative classes; (iii) it developed a stacking strategy to facilitate LRI prediction by integrating three individual classifiers. The results demonstrated that the stacking ensemble classifier can efficiently incorporate the advantages of each individual classifier; (iv) it utilized three distinct methodologies, namely, cell expression, expression product and specific expression, to comprehensively score CCC from multiple perspectives. It was easy to execute and did not require complex operations. As a result, SEnSCA obtained more accurate CCC analysis results, which were essentially consistent to those from CellPhoneDB, iTLAK, CellChat and CellComNet.

Although SEnSCA obtained strong performance on four benchmark datasets, it also had certain limitations. First, SEnSCA utilized SVM, 1‐DCNN and MHA for stacking. The Stacking model needs to combine multiple features when handling complex problems. However, as a supervised learning algorithm, it requires a large amount of labelled data for training. When labelled data are insufficient or difficult to annotate, the algorithm may not function well. Second, the SEnSCA stability was greatly enhanced by stacking even if a single base classifier performed poorly. However, its stability was also influenced by parameter selection and dataset features. When a dataset contains a large amount of noises or the data distribution changes, it may affect the model's performance. Lastly, SEnSCA used three base models for ensemble. Due to the requirement of more computational resources and time for ensemble learning, it may not be suitable for tasks requiring real‐time processing. Moreover, training and optimizing the model was more complex than optimizing a single model.

In addition, on dataset 1, SEnSCA computed an AUC of 0.8391 while CellComNet had an AUC of 0.8427, and its AUC was slightly lower than that of CellComNet. We considered it may be due to the following several factors. (i) Model architecture: SEnSCA was based on a combination of SVM, 1D‐CNN and MHA, while CellComNet utilized heterogeneous Newton gradient boosting algorithms and DNN. The architectural differences between the two models could lead to variations in their predictive capabilities. (ii) Feature representation: the feature representation approaches used by SEnSCA and CellComNet may capture different features of data, leading to different performance. And the features extracted by CellComNet were more discriminative for the specific characteristics of dataset 1, resulting in a slightly higher AUC. (iii) Data Distribution: the data distribution in dataset 1 may better favour the learning capabilities of CellComNet over SEnSCA. Differences in class balance, feature importance and noise levels could influence the models' performance. In summary, the slight difference in AUC between SEnSCA and CellComNet on dataset 1 could be attributed to a combination of factors related to model architecture, feature representation and data distribution.

The advancement of interaction prediction research in various fields of computational biology would provide valuable insights into genetic markers and ncRNAs related with cell–cell communication inference, such as miRNA‐lncRNA interactions,^44^ lncRNA‐disease associations,^45^ drug‐target interactions,^46^, ^55^ circRNA‐disease associations^47^ and metabolite‐disease associations.^48^ These association prediction models provide us many important references for LRI identification, and help to deeply understand the heterogeneity of cancers^49^, ^50^ and capture potential therapeutic targets.^51^, ^52^ Furthermore, oscillatory signals participate in numerous physiological processes.^53^ Caspase‐1 and GSDMD can induce the coexistence of pyroptosis and apoptosis of cells.^51^ ODE‐based theoretical modelling studies on gene/protein signalling networks have been equally important for the study of understanding regulatory mechanisms and finding potential therapeutic targets in diseases.^51^, ^52^, ^53^ Thus, we'll consider these signals in CCC prediction. More importantly, deep learning has powerful feature learning ability and has been widely applied to various classification tasks.^54^, ^55^, ^56^, ^57^ In the future, we will further boost the LRI identification performance by combining various association prediction models especially deep learning. Finally, spatial transcriptomics data provide abundant spatial context for each cell and help improve CCC inference. Thus, we will combine spatial transcriptomics data for CCC analysis.^58^, ^59^

## 5 CONCLUSION

In this study, we proposed SEnSCA, a framework for evaluating CCC. SEnSCA mainly contains potential LRI discovery and LRI‐mediated CCC inference. It better implemented LRI prediction by selecting negative LRI samples through a K‐means clustering and constructing a Stacking model that combines SVM, 1D‐CNN and MHA. Following LRI identification, CCC strength was evaluated by combining LRI filtering and scRNA‐seq data. We performed a few experiments for assessing the SEnSCA performance. Additionally, we visualized the constructed CCC network. The results demonstrated its ability to accurately infer CCC and construct a CCC network. Our findings highlight the strong associations between CAFs and melanoma cells.

In the future, we will try to employ more advanced optimization algorithms or more complex neural network architectures to enhance the LRI prediction performance. We will also intend to combine spatial transcriptomics data and statistical methods to construct a more efficient cellular communication inference framework. By analysing spatial transcriptomics data, we can gain a deeper understanding about communication patterns between cancer cells and normal cells within their tumour microenvironment. We anticipate that our proposed SEnSCA method can help us understand disease mechanisms more deeply and further discover new treatment strategies.

## AUTHOR CONTRIBUTIONS

**Liqian Zhou:** Conceptualization (equal); funding acquisition (equal); investigation (equal); methodology (equal); project administration (equal); validation (equal); writing – original draft (equal); writing – review and editing (equal). **Xiwen Wang:** Investigation (equal); methodology (equal); software (equal); writing – original draft (equal). **Lihong Peng:** Conceptualization (equal); investigation (equal); methodology (equal); project administration (equal); software (equal); validation (equal). **Min Chen:** Funding acquisition (equal); investigation (equal); project administration (equal); validation (equal); writing – review and editing (equal). **Hong Wen:** Funding acquisition (equal); investigation (equal); project administration (equal).

## FUNDING INFORMATION

This manuscript was supported by National Natural Science Foundation of China under Grant number 62072172.

## CONFLICT OF INTEREST STATEMENT

The authors declare no potential conflict of interests.

## Data Availability

Source codes and datasets are freely available at https://github.com/plhhnu/SEnSCA.
